# Effectiveness and feasibility of a mobile health self-management intervention in rheumatoid arthritis: study protocol for a pragmatic multicentre randomised controlled trial (AEGORA)

**DOI:** 10.1186/s13063-023-07733-y

**Published:** 2023-10-28

**Authors:** Michaël Doumen, Elias De Meyst, Cedric Lefevre, Sofia Pazmino, Johan Joly, Delphine Bertrand, Mieke Devinck, René Westhovens, Patrick Verschueren

**Affiliations:** 1 Department of Development and Regeneration, Skeletal Biology and Engineering Research Centre, KU Leuven, Leuven, Belgium; 2 Rheumatology, University Hospitals Leuven, Leuven, Belgium; 3 Rheumatology, AZ Sint-Lucas, Bruges, Belgium

**Keywords:** Rheumatoid arthritis, Self-management, Mobile health, Mobile applications, Telemedicine, Patient-reported outcomes, Randomised controlled trial

## Abstract

**Background:**

Rheumatoid arthritis (RA) considerably impacts patients’ lives. Patients’ confidence in their ability to manage this impact, or self-efficacy, can be supported with self-management interventions. One approach is to use mobile health (mHealth) applications, which can additionally provide insight into disease impact by remotely monitoring patient-reported outcomes. However, user engagement with mHealth-apps is variable, and concerns exist that remote monitoring might make patients overly attentive to symptoms.

**Methods:**

App-based Education and GOal setting in RA (AEGORA) is a multicentre, pragmatic randomised controlled trial investigating an mHealth-based self-management intervention to improve self-efficacy and remotely monitor disease impact in patients with RA. The intervention is provided via an adapted version of the application Sidekick (Sidekick Health, Reykjavik, Iceland) and consists of education, goal setting, lifestyle advice, and remote assessment of the Rheumatoid Arthritis Impact of Disease (RAID) questionnaire.

Across two centres, 120 patients will be recruited and randomised (2:1:1) to usual care or intervention group A/B (study app with weekly/monthly prompts to complete the RAID, respectively). Outcomes are assessed at baseline and after 4–6 months. The primary endpoint is a clinically important improvement (≥ 5.5/110) in the Arthritis Self-Efficacy Scale in the combined intervention group compared to usual care. Secondary endpoints are (a) non-inferiority regarding pain catastrophising, as a measure of symptom hypervigilance; (b) superiority regarding the RAID, sleep quality, and physical activity; and (c) participant engagement with the study app. Finally, the relationship between engagement, prompted frequency of RAID questionnaires, and the primary and secondary outcomes will be explored.

**Discussion:**

The AEGORA trial aims to study the effectiveness of mHealth-based, multicomponent self-management support to improve self-efficacy in the context of RA, while providing potentially valuable insights into temporal disease activity dynamics and the feasibility and possible negative effects of remote symptom monitoring in this population.

**Trial registration:**

Clinicaltrials.gov NCT05888181. Retrospectively registered on March 23, 2023. Study inclusion started on March 3, 2023.

**Supplementary Information:**

The online version contains supplementary material available at 10.1186/s13063-023-07733-y.

## Background

Rheumatoid arthritis (RA) is the most common form of chronic inflammatory arthritis, with an estimated prevalence of 0.5–1% [1]. RA usually presents with pain and swelling of the small joints and significantly impacts patients’ quality of life, physical functioning, and work participation [2]. Both the so-called treat-to-target strategy and novel disease-modifying antirheumatic drugs (DMARDs) have considerably improved outcomes for most patients with RA [3]. Nevertheless, many patients still experience ongoing pain and fatigue or suboptimal psychosocial wellbeing [4–6], while others are confronted with comorbidities that complicate the management of their disease [7, 8]. Consequently, it is becoming increasingly clear that managing RA requires a biopsychosocial approach, including attention for disease burden that is best addressed non-pharmacologically [9]. In part, this requires regular assessment of disease impact, for instance via patient-reported outcome (PRO) instruments, in order to highlight potential targets for further intervention.

In this regard, a crucial aspect of care is empowering patients to assume a more active role in the shared decision-making process, which is often termed self-management behaviour [10]. The benefits of self-management are clear on both the individual and the societal level. RA is a chronic disease with an often-unpredictable course, characterised by intermittent flares and even day-to-day symptom variation [11, 12]. Since most patients see their rheumatologist only 3–5 times per year on average [13], people living with RA should be supported in their ability to manage or react to flares, symptoms or other difficulties they encounter in between clinic visits [14, 15]. Consequently, self-management interventions have shown varying improvements in numerous health outcomes, while on a societal level, improved self-management might also reduce health care utilisation [16, 17]. A key concept regarding self-management is self-efficacy, or patients’ confidence in their ability to control the disease and its consequences, which has been shown to positively affect various aspects of living with RA [18, 19]. Self-efficacy is endorsed by the European Alliance of Associations for Rheumatology (EULAR) as both an important facilitator and an outcome of self-management interventions for inflammatory arthritis [20]. Indeed, several studies have shown that self-efficacy is not a static personality trait, but can be improved with personalised patient education and psychological support [21, 22].

In addition, EULAR defined several recommendations for the content of self-management interventions, including patient education and lifestyle advice, for example relating to physical activity, sleep, or diet [20]. A healthy lifestyle is not only relevant for the management of RA, but also to prevent and manage comorbidities like cardiovascular disease, which is more prevalent in patients with RA [23]. Finally, the EULAR recommendations highlight the potential benefits of using digital tools, such as mobile health (mHealth) applications, to facilitate these interventions [20]. Mobile applications can be a convenient and accessible way to support self-management in patients’ everyday environment, outside of the clinical office [24]. In addition, apps can be used to remotely monitor PROs, providing a more accurate picture of disease activity over time [25]. Indeed, studies based on various forms of digitally delivered self-management interventions for RA have shown promising results, for instance decreasing healthcare utilisation and improving empowerment, physical activity and hand functioning [26, 27]. Similarly, web-based educational programmes have proved effective in supporting self-efficacy among patients with RA in the past [28].

Nevertheless, providing self-management interventions for RA through mHealth raises several challenges. For one, studies have reported large variations in user engagement with mobile apps in clinical practice [29, 30]. While motivational principles like gamification are often proposed as potential solutions to this problem, most existing mHealth-apps do not include these features [31]. Secondly, qualitative studies have suggested that mHealth-apps might induce negative perceptions and anxiety by increasing patients’ focus on symptoms [32, 33]. To date, however, research regarding these effects remains scarce and conflicting [29]. Finally, the content of self-management interventions differs widely across published studies, most interventions are insufficiently supported by a theoretical framework, and mHealth remains underrepresented in the literature as an approach to deliver self-management interventions for inflammatory arthritis [34].

We hereby provide the protocol for a multicentre, pragmatic randomised controlled trial (RCT) studying the effectiveness and feasibility of an mHealth-based self-management intervention aiming to improve self-efficacy for the management of RA-related symptoms. The intervention consists of education, lifestyle advice and remote monitoring elements supported by motivational features and gamification, and is based on principles of goal setting, self-efficacy theory and behavioural economics. As a key secondary objective, the trial aims to assess if such an intervention is associated with changes in pain catastrophising, as a measure of hypervigilance to symptoms.

## Methods and analysis

### Study setting

App-based Education and GOal setting in Rheumatoid Arthritis (AEGORA) is a pragmatic, multicentre RCT conducted in a superiority setting with 2:1:1 allocation to either usual care or one of two different versions of an mHealth-based self-management intervention for RA. The trial will be conducted in two hospitals across Belgium: an academic centre, University Hospitals Leuven, and a non-academic hospital, AZ Sint-Lucas Bruges. Both hospitals have a strong teaching background, ample experience in the management of RA, and a shared electronic medical record (EMR) system.

### Study population and recruitment

Consecutive patients will be assessed for eligibility by their treating rheumatologist during outpatient clinic visits in the Rheumatology department of both participating centres. Patients will be considered eligible if they:

- ▪ Provide written informed consent for participation.
- ▪ Are 18 years of age or older.
- ▪ Were diagnosed with RA by a rheumatologist minimally 16 weeks before. This time frame was chosen based on previous work of our research group, which suggested that the dynamic first weeks after diagnosis are less suitable to assess psychosocial outcomes [4].
- ▪ Are able to understand and read Dutch.
- ▪ Have access to a smartphone with a recent operating system and feel comfortable using it.

To include an optimally representative patient population, no additional exclusion criteria will be applied for this study. When the treating rheumatologist considers a patient to be eligible, a researcher will verify eligibility before reviewing and signing the informed consent form (ICF) with the patient. Patients who agree to participate but do not meet the inclusion criteria upon verification will be considered screen failures. In these cases, only age, sex, disease duration, and the reason for screen failure will be collected. For patients who refuse to participate, reasons for non-participation will be collected, when disclosed.

### Study intervention

Participants will be randomised to one of two intervention groups (A/B) or to usual care, which includes a standardised educational leaflet about RA. The study intervention consists of a self-management programme accessible via the smartphone application Sidekick, developed by software company Sidekick Health (Reykjavik, Iceland) and adapted to two different study versions (A/B) in collaboration with the research team.

The study app’s content is based on the self-efficacy theory with elements from behavioural economics [18, 35]. Overall, the programme aims to improve self-efficacy by providing individuals with tailored information and achievable goals that help them build confidence. Moreover, users are provided feedback and encouragement in the form of personalised messages and a points-based gamification system.

**Fig. 1:**
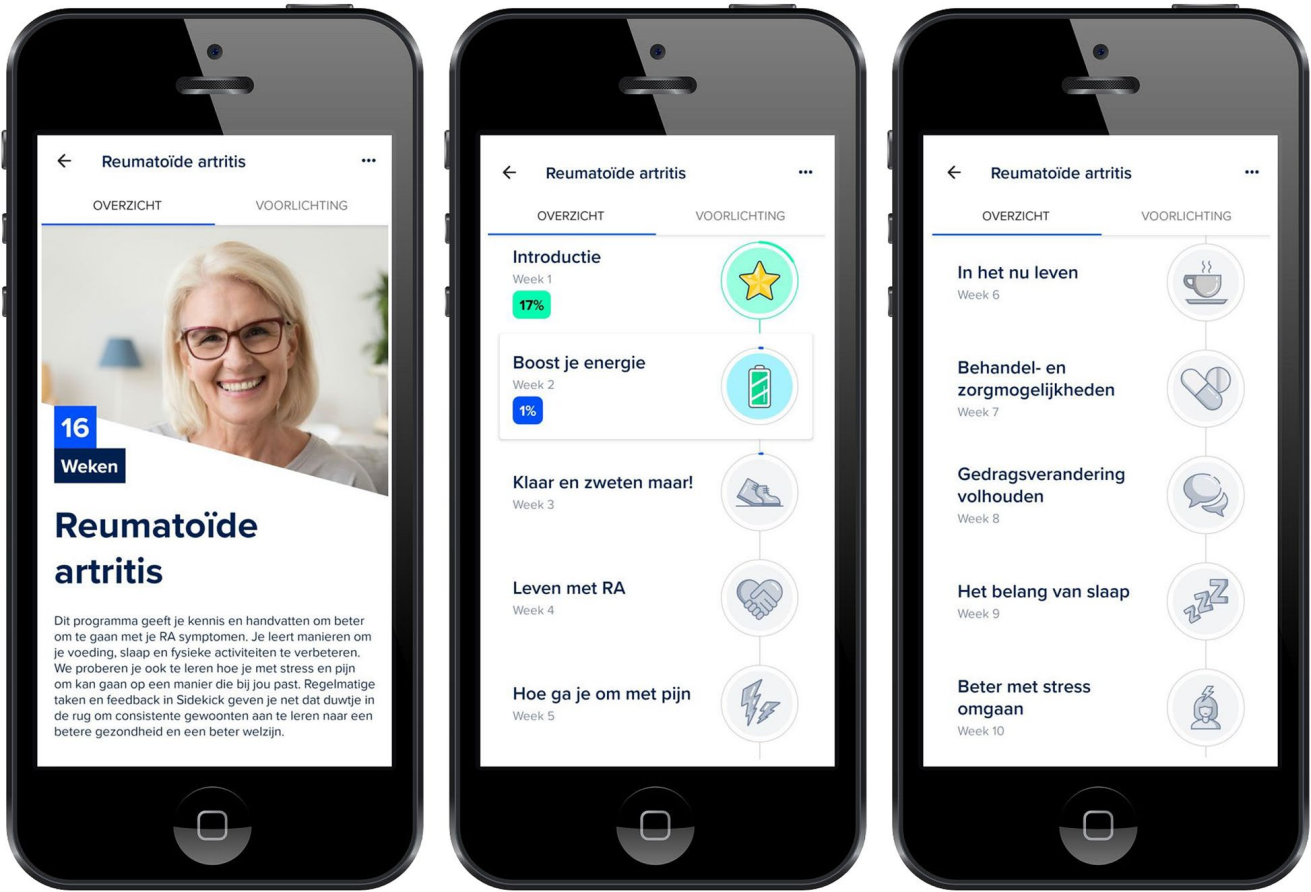
Overview of the educational programme as presented in the study app

**Fig. 2:**
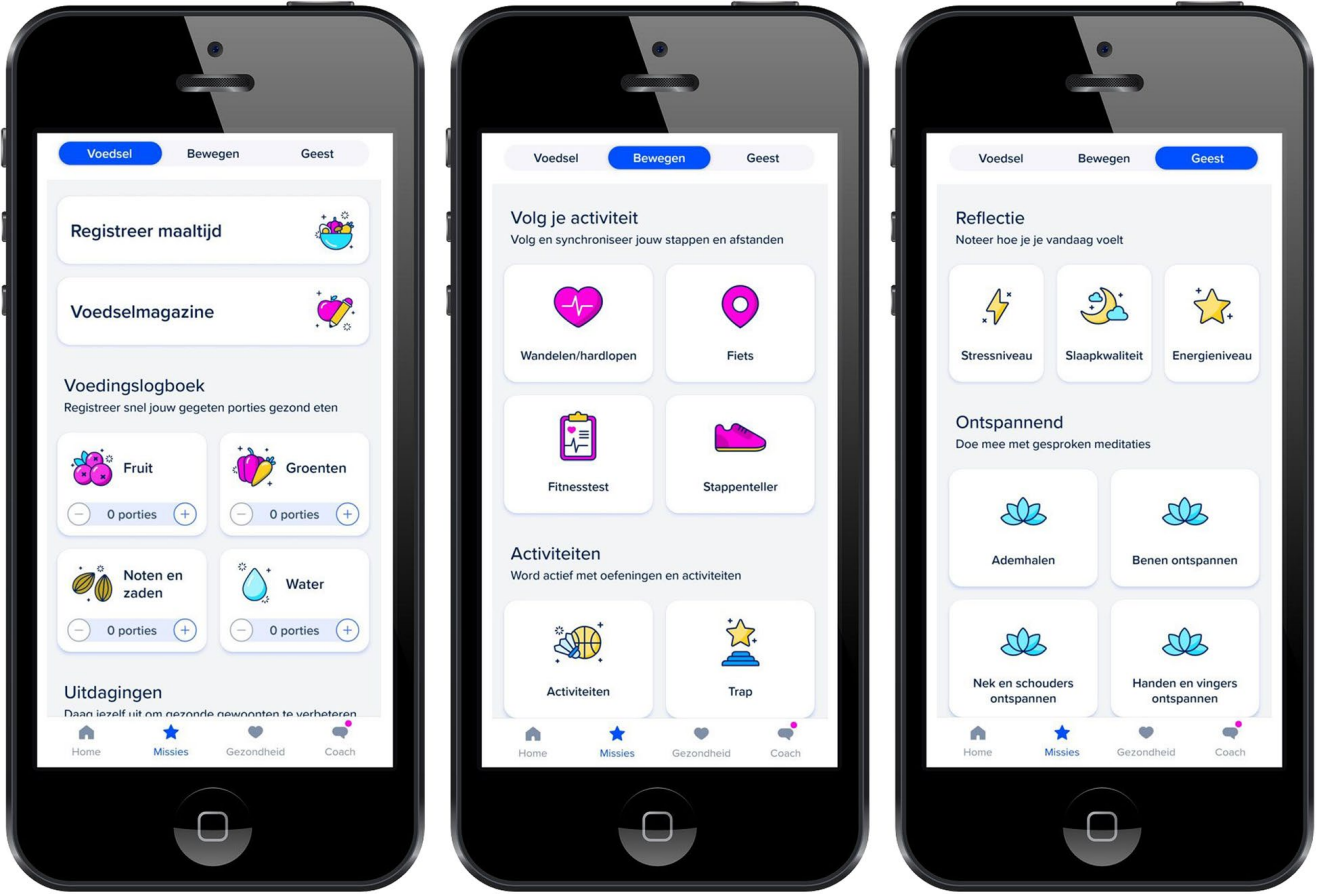
Overview of the diary functions to monitor diet, physical activity and mental health in the study app

The study app’s programme comprises several components to support self-management. First, the app contains an RA-specific educational programme consisting of 16 weekly modules. Each module relates to a different topic regarding living with RA, presented as sequential videos at prespecified time points (Fig. 1). The educational content was co-developed by a panel of rheumatologists and patient research partners (PRPs). Second, the app provides patients with tailored lifestyle advice, both as part of the educational programme and in the form of personalised messages from a certified health coach employed by the app developer. Specifically, general information is provided to all users via the app regarding the benefits of physical activity, a balanced diet and a regular sleeping pattern, while more personalised, non-medical information can be additionally communicated by the health coach based on participants’ activity in the app. Third, the study app includes remote monitoring features underpinned by goal-setting principles. For instance, participants can use the app to log daily steps, physical activity, diet, sleep, and mental health (Fig. 2). Personal goals relating to these aspects, as well as physical and mental challenges and exercises, can then be set up to encourage behavioural change.

For the purpose of this study, several modifications to the app were made in collaboration with the research team. Specifically, interaction with the health coach was limited to one-way communication to ensure a more comparable, albeit individually tailored, provision of information. Additionally, patient-reported disease impact can be monitored within the app as part of the study via prompted completion of the Rheumatoid Arthritis Impact of Disease (RAID) instrument [36]. Based on random allocation to one of both intervention groups, the RAID will be prompted either weekly (group A) or monthly (group B), to explore the influence of requested PRO-reporting frequency on specific outcomes as described below.

### Study objectives

#### Primary objective

To assess whether patients with RA experience an improvement in arthritis-related self-efficacy after an mHealth-based self-management intervention consisting of disease education, goal setting, lifestyle advice, and remote monitoring features.

#### Secondary objectives

- ▪ To investigate in a non-inferiority setting if the study intervention leads to changes in pain catastrophising, a more negative cognitive-affective appraisal of symptoms, and if these changes are influenced by PRO-reporting frequency.
- ▪ To assess whether the study intervention leads to changes in physical activity, sleep quality, or patient-reported disease impact.
- ▪ To explore the feasibility and usefulness of remotely monitoring patient-reported disease impact over time.
- ▪ To describe participants’ engagement with the study app, predictors of engagement (including requested PRO-reporting frequency) and the relationship between engagement and the study outcomes.

### Trial procedures and randomisation

All participants will have a baseline clinic visit and a follow-up visit, which will be scheduled as in routine care but should be minimally 4 and maximally 6 months from baseline. Table 1 presents the outcomes to be collected at each visit. Clinical and laboratory examinations are performed as in usual care. Before each visit, patients complete a number of PRO measures (Table 1) that are routinely collected in clinical practice. These questionnaires are completed online via the hospital’s EMR companion platform, or via a tablet or in pen-and-paper form in the clinic waiting room. After signing the ICF, participants will additionally complete a number of study-specific outcome measures (Table 1) at both the baseline and follow-up visit within the web-based platform Research Electronic Data Capture (REDCap), using a unique QR-code or email link [37]. If the study questionnaires are not completed after 3 days, a reminder will be sent via e-mail. If the questionnaires are not completed by 1 week after the study visit, participants will be contacted via telephone by the research team.

**Table 1:** Procedures and outcome measures (SPIRIT table)

	**Baseline**	**During intervention**	**Follow-up** **(4–6 months)**
**Procedure/outcome measure**			
Eligibility screening	**♦**		
Informed consent	**♦**		
Randomisation/allocation	**♦**		
Set-up and installation of study app (in intervention groups)	**♦**		
**Demographic/clinical characteristics**			
Age (years)	**♦**		
Sex (male/female)	**♦**		
Body mass index (kg/m^2^)	**♦**		
Lifestyle (smoking, alcohol use)	**♦**		
RF and ACPA status (positive/negative)	**♦**		
Erosive changes on latest radiograph (yes/no)	**♦**		
Disease duration (months/years)	**♦**		
Current and prior DMARD therapy and glucocorticoid/analgesic intake	**♦**		**♦**
Comorbidities and extra-articular manifestations	**♦**		
**Outcome measures as part of routine care**			
Patient global assessment of disease activity (VAS)	**♦**		**♦**
Physician global assessment of disease activity (VAS)	**♦**		**♦**
Tender joint count and swollen joint count (0–28)	**♦**		**♦**
C-reactive protein (mg/L)	**♦**		**♦**
Erythrocyte sedimentation rate (mm/h)	**♦**		**♦**
Nocturnal pain (yes/no)	**♦**		**♦**
Morning stiffness (yes/no)	**♦**		**♦**
HAQ-DI (0–3)	**♦**		**♦**
**Study-specific outcome measures**			
RAID (0–10)	**♦**		**♦**
- Intervention group A: weekly		**♦**	
- Intervention group B: monthly		**♦**	
ASES (11–110)	**♦**		**♦**
PCS (0–52)	**♦**		**♦**
IPAQ-S (METs per week)	**♦**		**♦**
PSQI (0–21)	**♦**		**♦**
In-app logged usage data^a^		**♦**	
Educational level (primary/secondary/Bachelor’s/Master’s/doctoral)	**♦**		
Prior experience with mHealth (yes/no)	**♦**		
Preference to continue using app after study (yes/no)			**♦**
Satisfaction with intervention (VAS)			**♦**

*RF* rheumatoid factor, *ACPA* anti-citrullinated peptide antibodies, *DMARD* disease-modifying antirheumatic drug, *VAS* visual analogue scale, *HAQ-DI* Health Assessment Questionnaire Disability Index, *RAID* Rheumatoid Arthritis Impact of Disease, *ASES* Arthritis Self-Efficacy Scale, *PCS* Pain Catastrophizing Scale, *IPAQ-S* International Physical Activity Questionnaire Short form, *METs* Metabolic Equivalents of Task, *PSQI* Pittsburgh Sleep Quality Index

^a^Collected at study end in pseudonymised form. Usage data concern physical activity (including daily step count), diet, stress management, goals set, videos accessed, logins, and completed questionnaires

In addition, patients’ demographic and clinical characteristics will be entered into an electronic case report form (eCRF) within REDCap. Participants are then randomised (2:1:1) to either usual care, intervention group A or intervention group B (study app with weekly or monthly prompts to complete the RAID, respectively). Randomisation is performed with a designated module in REDCap, based on (local) randomisation stratified by the study centre. The random allocation sequence was computer-generated to ensure allocation concealment.

The control group will be followed according to usual care standards. This includes informal screening for general wellbeing during outpatient clinic visits, with referral to specific allied health professionals for additional education or non-pharmacological support if needed. As part of standard care, participants in both the control group and the intervention groups will also receive a standardised educational leaflet about RA.

In addition to usual care, participants in the intervention group will receive access to the study app as described above. To ensure the protection of participants’ personal data, a pseudonymised study account will be created for each participant randomised to the intervention. This study account is based on the participant’s unique study ID and is used to log into the study app. After the final study visit, the account is terminated, and all personal data connected to it is removed from the application’s data cloud. However, study participants in both the control and intervention groups will be free to use the Sidekick application free of cost after the study.

### Outcome measures

Table 1 provides an overview of all outcomes, both standard care and study-specific, that will be collected during the trial, as well as the timing of their assessment (at baseline, at the follow-up visit after 4–6 months, and/or via the study app in-between these visits). The following outcomes are study-specific measures to be collected:

- ▪ Self-efficacy for arthritis-related symptoms, measured with the Arthritis Self-Efficacy Scale (ASES) [38]. The ASES is a patient-reported questionnaire consisting of 20 items across 2 subscales: self-efficacy for managing pain (range 5–50) and other symptoms (range 6–60). Both scores can be summed to derive a total ASES score (range 11–110). Higher scores indicate higher perceived self-efficacy.
- ▪ Impact of RA on various aspects of life, measured with the RAID [36]. The RAID consists of 7 items on a 0–10 numeric rating scale, enquiring about the impact of RA on pain, functional limitations, fatigue, sleep, physical wellbeing, emotional wellbeing, and coping. Higher scores indicate more perceived disease impact. The RAID will be collected at both study visits and via the study app in between visits on either a weekly or monthly basis (intervention groups A and B, respectively).
- ▪ Cognitive-affective appraisal of pain, measured with the Pain Catastrophizing Scale (PCS) [39]. The PCS comprises 13 items on a 0–4 Likert scale, resulting in a total score of 0–52 with subscales for rumination, magnification, and helplessness. Higher scores indicate more catastrophic perceptions concerning pain.
- ▪ Physical activity, measured with the International Physical Activity Questionnaire Short form (IPAQ-S) [40]. The IPAQ-S is a 7-item questionnaire regarding physical activities during the last 7 days. An activity score is obtained for different domains, each multiplied with the accompanying metabolic equivalent of task (MET) value, leading to a sum score corresponding with low, moderate, or high physical activity.
- ▪ Patient-perceived sleep quality, measured with the Pittsburgh Sleep Quality Index (PSQI) [41]. The PSQI measures sleep quality through 19 items across 7 domains, with a resulting total score ranging from 0 to 21 and higher scores reflecting worse sleep quality.
- ▪ Participants’ educational level and prior experience with mHealth-apps will be collected at the baseline visit.
- ▪ Upon completion of the study, we will assess the participants’ overall satisfaction with the intervention on a visual analogue scale and enquire if they would like to continue using the app.
- ▪To assess feasibility, pseudonymised app usage data are passively collected and provided to the study team by Sidekick Health upon study completion. Usage data include registered information concerning diet, stress management, goals set, videos accessed, logins, completed questionnaires, and physical activity (including daily step counts registered through the smartphone’s accelerometer, if users choose to activate this function).

### Sample size

We hypothesise that the intervention is superior to usual care with respect to self-efficacy improvement. Sample size calculation was based on a minimal clinically important difference (MCID) in the ASES score of 5.5 [21, 42]. Moreover, data from an early RA study at the host institution showed a mean (± SD) self-efficacy for pain and other symptoms of 31.8 (± 8.9) and 42.6 (± 9.4), respectively [43]. Given this information, an effect size of approximately 0.59 would correspond with a clinically important difference of 5.5 from the expected population mean. Following these assumptions, 94 participants are needed to detect a clinically meaningful difference in the ASES score with 80% power and a significance level of 0.05. Based on previous research and outpatient clinic attendance experience, we increased the intended sample size with an expected dropout rate of 10% [21, 30, 44]. Furthermore, the ASES was not normally distributed in the prior study. Following a general rule of thumb, the sample size was thus further increased by 15% to account for the loss of power non-parametric tests would imply [45]. Consequently, a total of 120 participants will be recruited.

An additional sample size calculation was conducted for the main secondary objective, comparing the PCS score between intervention and control groups. Based on pooled data from the PCS development/validation studies and a French RA cohort, the population-weighted mean (SD) PCS score is estimated at 20.3 (SD 12.3) [39, 46, 47]. Thus, an MCID on the PCS, proposed as greater than 38% change [39, 48], would correspond to ≥ 7.7/52. To exclude effects of the intervention on pain catastrophising, we chose a non-inferiority design for this outcome with the aforementioned MCID of 7.7 as the non-inferiority margin. Non-inferiority will thus be confirmed if the upper bound of the 95% CI for the intervention’s effect on PCS remains within a clinically important margin [49]. Following these assumptions and allowing for a 10% dropout rate, a total of 82 participants need to be included to demonstrate non-inferiority of the intervention regarding the PCS score with 80% power, a minimal clinically important margin and a two-sided significance level of 0.025. When 120 patients are included as per the primary outcome, and assuming a 10% drop-out rate, we should have 90.2% power to demonstrate non-inferiority for this secondary outcome.

All sample size calculations were conducted via R version 4.2.1, using the packages *pwr* and *epiR.*

### Study endpoints

#### Primary endpoint

The primary endpoint is defined as achieving an improvement (increase) of 5.5 (MCID) in the total ASES score at the follow-up visit in favour of the intervention group (groups A and B combined) when compared to the control group.

#### Secondary endpoints

- ▪ Non-inferiority of the study intervention (groups A and B combined) compared with standard care regarding change from baseline in the PCS total score at follow-up. Additionally, a post-hoc analysis will be carried out comparing the PCS between intervention groups A and B, to study the influence of PRO-reporting frequency on pain catastrophising.
- ▪ Superiority of the study intervention over standard care regarding the change from baseline in the IPAQ-S sum score, the PSQI total score, and the RAID at the follow-up visit.
- ▪ Feasibility of the study intervention in terms of user engagement, described as the proportion of completed RAID questionnaires in the study app, app usage data, and the influence of PRO-reporting frequency on these outcomes (intervention group A versus B). Additionally, post-hoc analyses will be carried out to study the relationship between user engagement and the primary and other secondary outcomes.

### Statistical analysis

Statistical analyses will be carried out in R. Missing data will be handled with multiple imputation when data can be assumed to be missing at random. Correction for multiple testing will be applied where appropriate.

Descriptive statistics will be reported for relevant population characteristics at baseline (Table 1). These characteristics will be presented for the total study population and for the control and intervention groups separately.

All analyses will be carried out in the intention-to-treat (ITT) population, consisting of all patients who completed the ASES at baseline and either installed the study app (intervention groups) or were assigned to the control group. Additionally, sensitivity analyses will be carried out in the per-protocol (PP) population, consisting of only those patients who attended the follow-up visit and completed ≥ 50% of in-app RAID questionnaires. Finally, should there be important differences in potential confounders between groups despite randomisation, we will perform a sensitivity analysis adjusting for these differences.

#### Primary endpoint

The total ASES score at the follow-up visit will be compared between the control and total intervention group (A and B combined) using analysis of covariance (ANCOVA) adjusted for baseline scores.

#### Secondary endpoints

- ▪ The PCS score will be compared between the control and total intervention group at the follow-up visit using ANCOVA adjusted for baseline values, in a non-inferiority setting. As a post-hoc analysis, we will additionally compare the PCS score between intervention groups A and B using a similar method, to study the effect of PRO-reporting frequency.
- ▪ The IPAQ-S, PSQI and RAID scores will be compared between the control and total intervention group at the follow-up visit using ANCOVA adjusted for baseline values.
- ▪ Participants’ engagement with the study app will be summarised descriptively, based on the proportion of prompted RAID questionnaires that were completed and on in-app logged usage data (Table 1). Additionally, we will compare engagement outcomes between intervention groups A and B with an unpaired *t*-test or Mann–Whitney *U*-test depending on data distribution, to study the effect of PRO-reporting frequency. Finally, post-hoc analyses will be carried out to explore the relationship between participant engagement and the primary and other secondary outcomes, using linear regression adjusting for clinically relevant covariates.

### Ethics and dissemination

This trial will be conducted in compliance with the Declaration of Helsinki, the principles of Good Clinical Practice and all applicable regulatory requirements. The study was approved by the Ethics Committee Research of the University Hospitals Leuven (reference S66633) after consultation of the committee at AZ Sint-Lucas Bruges. Progress reports for the trial will be provided to the Ethics Committee on a yearly basis by the research team and are mandatory to retain ethical approval for the study. Study results will be disseminated via conferences, publications in peer-reviewed journals and through patient organisations.

This trial does not involve an investigational medicinal product and has no influence on participants’ pharmacological treatment. Similarly, no additional laboratory investigations will be performed other than those needed for usual care. Consequently, we expect no adverse events (AEs) directly related to the intervention, and no Data Monitoring Committee was formed for this low-risk intervention. Treatment-related complications or AEs are registered and reported as part of routine care.

Data monitoring will be performed by the research team at University Hospitals Leuven on a regular basis via monitoring of the eCRF and in accordance with Good Clinical Practice guidelines and local regulations. All study data are stored and archived for at least 20 years in a secure environment hosted by University Hospitals Leuven. Sidekick Health has access to no information other than the pseudonymised app usage data, which are stored in their Google Cloud SQL service until deletion of the study account at completion or discontinuation of the trial. Google does not have access to this information for any other reason than to store it. Data storage and maintenance are in accordance with Belgian and European legal requirements and with the European General Data Protection Regulation.

### Patient and public involvement

Both the rationale for the trial and the choice of study outcomes were based on previously published qualitative research conducted at the host institution among patients with RA. These studies investigated patient-preferred outcomes of RA treatment and specific barriers and facilitators for mHealth use [32, 50]. PRPs were involved in both the setup and analysis of these studies and in the adaptation of the study app’s educational content. Finally, a summary report of the study results will be disseminated via the publications of the Belgian rheumatology patient organisation ReumaNet.

## Discussion

Recent evidence supports that adequate management of RA should go beyond pharmacological treatment alone. Given RA’s nature as a lifelong disease with temporal variability in symptoms, patients should be supported in their ability to self-manage the disease’s consequences in their daily lives. By using an mHealth approach, AEGORA aims to offer multicomponent self-management support to patients in their own preferred time and space. The self-management intervention consists of education, lifestyle support, goal setting and personalised coaching, all of which are relevant and potentially effective ways to improve patients’ self-efficacy [17]. Moreover, the trial could provide valuable insight into disease activity dynamics by remotely monitoring patient-reported disease impact.

Since many patients with RA continue to experience an impact of the disease even when disease activity is well-controlled [6], there is a pressing need for accessible means to support patients in their self-management of this impact. Despite their promise in this context, digital tools are still relatively underrepresented in the current literature regarding self-management interventions for RA [17, 34]. The AEGORA trial’s results could help to address the question of whether mobile applications can serve as a valuable resource for both physicians and other healthcare providers to improve self-management confidence and to gain more insight into patients’ perceived disease impact.

However, despite its potential advantages, using mHealth to deliver self-management interventions for RA raises its own specific issues, including the well-established challenge of ensuring durable user engagement with smartphone apps [29]. Moreover, qualitative studies have suggested a possible negative effect of mHealth interventions on patients’ perceptions regarding illness and symptoms [32, 51], although quantitative evidence to support these concerns remains limited and conflicting [52, 53]. AEGORA aims to mitigate these challenges by including motivational elements and gamification principles in the study app, and by examining as a key secondary outcome if the intervention might have a negative effect on pain catastrophising. Moreover, the study intervention is supported by a relevant theoretical background in psychology and by prior qualitative studies [32, 50]. Finally, the educational content was co-developed by rheumatologists and PRPs, and the trial will be conducted in both an academic and non-academic setting.

In addition to these strengths, the trial design raises some limitations. First, as with any multicomponent intervention, it will be difficult to ascertain which components are responsible for any positive effects that might be shown. Second, owing to the limited duration of follow-up, this trial will not be able to provide information on long-term effectiveness. Since the timing of follow-up visits was intentionally kept close to routine care, the trial’s duration may also vary from 4 to 6 months across participants. Finally, despite our aim to approach all potentially eligible patients, we expect a higher response rate in patients who are more experienced with digital technologies. Although this limitation is inherent to any mHealth intervention, it could reduce the generalisability of the trial’s results. Exclusion criteria were therefore limited to allow for the inclusion of patients with a wide range of clinical and demographic characteristics.

In conclusion, the AEGORA trial aims to study the effectiveness of mHealth-based, multicomponent self-management support to improve self-efficacy in the context of RA, while providing potentially valuable insights into temporal disease activity dynamics and the feasibility and possible negative effects of remote symptom monitoring in this population.

## Trial status

Version 6.3 of the trial protocol was approved by the Ethics Committee Research of the University Hospitals Leuven (reference S66633) on November 4, 2022, after consultation of the committee at AZ Sint-Lucas Bruges. Recruitment for the trial commenced on March 3, 2023, and is expected to be completed by the end of September 2023 with an estimated study completion by March 2024.

### Supplementary Information

**Additional file 1.** Reporting checklist for protocol of a clinical trial.

** Additional file 2.** Informed consent form.

## Data Availability

Data relating to this study protocol, the statistical code and the protocol itself are available from the corresponding author upon reasonable request. All study data are stored and archived for at least 20 years in a secure environment hosted by University Hospitals Leuven. Sidekick Health has access to no information other than the pseudonymised app usage data, which are stored until completion or discontinuation of the trial. Study results will be disseminated via conferences, publications in peer-reviewed journals and through patient organisations.

## Abbreviations

RA: Rheumatoid arthritis
mHealth: Mobile health
AEGORA: App-based Education and GOal setting in RA
DMARD: Disease-modifying antirheumatic drug
PRO: Patient-reported outcome
EULAR: European Alliance of Associations for Rheumatology
EMR: Electronic medical record
ICF: Informed consent form
PRP: Patient research partner
RCT: Randomised controlled trial
RAID: Rheumatoid Arthritis Impact of Disease
REDCap: Research Electronic Data Capture
eCRF: Electronic case report form
ASES: Arthritis Self-Efficacy Scale
PCS: Pain Catastrophising Scale
IPAQ-S: International Physical Activity Questionnaire Short form
PSQI: Pittsburgh Sleep Quality Index
MCID: Minimal clinically important difference
ITT: Intention to treat
PP: Per protocol
ANCOVA: Analysis of covariance
AE: Adverse event
