# Supplementary material for: Effectiveness and feasibility of a mobile health self-management intervention in rheumatoid arthritis: study protocol for a pragmatic multicentre randomised controlled trial (AEGORA)
Source: Trials. 2023 Oct 28;24:697. doi: 10.1186/s13063-023-07733-y (PMC10613379; doi:10.1186/s13063-023-07733-y)
Supplement: Supplementary file 2 — Additional file 2. Informed consent form. [file 13063_2023_7733_MOESM2_ESM.pdf]

Titel van de studie: De invloed van ziekte-educatie en doelgericht levensstijladvies, aangeboden via een mobiele app, op zelfredzaamheid en een gezonde levensstijl bij patiënten met reumatoïde artritis.

Opdrachtgever: UZ Leuven/KU Leuven, Herestraat 49, 3000 Leuven

Onderzoeksinstelling: UZ Leuven/KU Leuven & AZ Sint-Lucas Brugge

Comité voor Medische Ethiek: Ethisch comité onderzoek UZ/KU Leuven

Lokale artsen-onderzoekers: prof. dr. Patrick Verschueren (UZ Leuven) & dr. Mieke Devinck (AZ Sint-Lucas Brugge)

Noodzakelijke informatie voor uw beslissing om deel te nemen

Inleiding[1]

U wordt uitgenodigd om deel te nemen aan een klinische studie. Het doel van deze studie is het evalueren van een bijkomende ondersteuning van uw behandeling voor reumatoïde artritis, en dit onder vorm van een mobiele applicatie (Sidekick). Deelname aan deze studie heeft geen invloed op andere aspecten van uw behandeling, zoals de medicatie die u ontvangt.

De Sidekick-app is bedoeld als een hulpmiddel om zo goed mogelijk om te gaan met uw ziekte in het dagelijkse leven, wat we soms ook “zelfmanagement” noemen. Zo biedt de app heel wat extra informatie en kennis over de ziekte in de vorm van 16 leermodules, die specifiek door reumatologen samengesteld werden. Via deze modules geeft Sidekick u ook doelgericht advies rond een gezonde levensstijl en de stappen die u zelf kan ondernemen om de controle over uw ziekte maximaal in handen te nemen. Naast het volgen van dit educatieve programma zal u in de app op vrijwillige basis ook relevante aspecten uit uw dagelijkse leven kunnen registreren, zoals uw fysieke activiteiten, stressniveau, pijn, slaapkwaliteit of het moment waarop u medicatie moet innemen. Al deze functies worden in de Sidekick-app gebundeld op een zo efficiënt mogelijke manier, dankzij het toepassen van motivatietechnieken en spelelementen (of “gamification”).

Het doel van deze klinische studie is na te gaan of het gebruik van deze mobiele app een positieve invloed kan uitoefenen op zelfredzaamheid, lichaamsbeweging en slaapkwaliteit bij patiënten met reumatoïde artritis.

Er is evenwel geen garantie dat uw deelname aan deze studie u voordeel zal opleveren. Voordat u beslist over uw deelname aan deze studie willen we u wat meer informatie geven over wat dit betekent op organisatorisch vlak en wat de eventuele voordelen en risico's voor u zijn. Zo kan u een beslissing nemen op basis van de juiste informatie. Dit wordt "geïnformeerde toestemming" genoemd.

Wij vragen u de volgende pagina’s met informatie aandachtig te lezen. Hebt u vragen, dan kan u terecht bij de arts-onderzoeker of zijn of haar vertegenwoordiger. Dit document bestaat uit 3 delen: essentiële informatie die u nodig heeft voor het nemen van uw beslissing, uw schriftelijke toestemming en bijlagen waarin u meer details terugvindt over bepaalde onderdelen van de basisinformatie.

Als u aan deze klinische studie deelneemt, dient u het volgende te weten:

[1] Een beschrijving en de resultaten van deze klinische studie zullen beschikbaar zijn via het internet (website EMA https://www.clinicaltrialsregister.eu/ ; FDA http://www.clinicaltrials.gov/) en gepubliceerd worden in medische tijdschriften.

- Deze klinische studie wordt opgestart na evaluatie door meerdere ethische comités.
- Uw deelname is vrijwillig; er kan op geen enkele manier sprake zijn van dwang. Voor deelname is uw ondertekende toestemming nodig. Ook nadat u hebt getekend, kan u de arts-onderzoeker laten weten dat u uw deelname wilt stopzetten. De beslissing om al dan niet (verder) deel te nemen zal geen enkele negatieve invloed hebben op de kwaliteit van de zorgen noch op de relatie met de behandelende arts(en).
- De gegevens die in het kader van uw deelname worden verzameld, zijn vertrouwelijk. Bij de publicatie van de resultaten is uw anonimiteit verzekerd.
- Er worden u geen kosten aangerekend voor specifieke behandelingen, bezoeken / consultaties, onderzoeken in het kader van deze studie.
- Er is een verzekering afgesloten voor het geval dat u schade zou oplopen in het kader van uw deelname aan deze klinische studie.
- Indien u extra informatie wenst, kan u altijd contact opnemen met de arts-onderzoeker of een medewerker van zijn of haar team.

Aanvullende informatie over “Rechten van de deelnemer aan een klinische studie” vindt u in bijlage 1.

Doelstelling en beschrijving van het studieprotocol

Wij nodigen u uit om deel te nemen aan een klinische studie inzake het gebruik van de Sidekick-app bij ca. 120 Belgische deelnemers.

Het doel van deze studie is te onderzoeken of educatieve ziekte-informatie en doelgericht levensstijladvies, aangeboden via de Sidekick-app, invloed heeft op zelfredzaamheid, lichaamsbeweging en slaapkwaliteit bij mensen die lijden aan reumatoïde artritis.

Daarnaast trachten de onderzoekers ook een beter beeld te krijgen van hoe de symptomen van reumatoïde artritis, geregistreerd in de app, evolueren of schommelen doorheen de tijd. Ten slotte zal onderzocht worden of het gebruik van de app ook invloed heeft op de manier waarop pijn beleefd wordt, en of dit afhankelijk is van hoe vaak de app gebruikt wordt.

Om aan het onderzoek deel te nemen wordt verwacht dat u aan volgende inclusiecriteria voldoet:

- U bent 18 jaar of ouder.
- U lijdt aan reumatoïde artritis, vastgesteld door een reumatoloog, en bent al minstens 4 maanden in behandeling.
- U of uw wettelijke gemachtigde vertegenwoordiger verstrekken vóór de screeningprocedure vrijwillige schriftelijke geïnformeerde toestemming voor elke activiteit gerelateerd aan de studie.
- U bent in staat om Nederlands te begrijpen en schrijven.
- U bent in het bezit van een smartphone met een Android (vereist Android 7.0 of later) of Apple iOS (vereist iOS 13.0 of later) besturingssysteem.

In dit onderzoek zullen twee groepen met elkaar worden vergeleken. Wanneer u instemt om deel te nemen, zal aan de hand van een computersysteem op willekeurige basis (randomisatie) worden bepaald tot welke studiegroep u toetreedt. Wanneer u tot de “controlegroep” behoort zal u de zorg krijgen volgens alle gebruikelijke standaarden

en krijgt u bijkomend ook een educatieve brochure over reumatoïde artritis, maar geen toegang tot de Sidekick-app. Wanneer u in de "interventiegroep" ingedeeld wordt, krijgt u naast de gebruikelijke zorg en de educatieve brochure bijkomend ook toegang tot de Sidekick-app.

Verloop van de studie

Uw deelname aan de studie neemt ongeveer 16 weken in beslag en omvat twee fysieke visites: aan het begin van de studie (week nul) en op week 16 (met een marge van 2 weken). Deze visites vallen gewoonlijk samen met uw gebruikelijke consultatiemomenten, zodat uw deelname geen extra bezoeken zal vereisen.

Patiënten met reumatoïde artritis die wensen deel te nemen aan de studie en voldoen aan de inclusiecriteria worden gevraagd om de "geïnformeerde toestemming" te ondertekenen. Zoals hoger beschreven worden de deelnemers vervolgens op willekeurige basis onderverdeeld in hetzij een "controlegroep", hetzij een "interventiegroep".

Deelnemers in beide groepen krijgen bij de start van de studie een informatiebrochure over reumatoïde artritis. Aan deelnemers in de interventiegroep wordt bijkomend gevraagd de Sidekick-app te downloaden - uit hetzij de Apple App Store of Google Play Store - en de algemene voorwaarden van Sidekick Health te accepteren.

Bij indeling in de interventiegroep zal de arts-onderzoeker tijdens de eerste studievisite de app samen met de deelnemer installeren en de praktische gebruiksinformatie overlopen.

In de loop van de studie beogen de onderzoekers volgende gegevens te verzamelen:

- Persoonlijke gegevens van de deelnemer zullen worden verzameld aan de hand van het medische dossier.

- Tijdens de twee studievisites zullen naast de standaardonderzoeken (zoals de gebruikelijke bloedafname) ook enkele korte vragenlijsten afgenomen worden. Deze vragenlijsten worden hetzij afgenomen op papier, hetzij via een QR-code, of via de myNexuz-applicatie naargelang uw voorkeur. De vragen peilen naar ziekte-impact, zelfredzaamheid, de manier waarop pijn beleefd wordt, fysieke activiteit en slaapkwaliteit, en nemen doorgaans maximaal 20 minuten in beslag.

- Aan deelnemers in de interventiegroep zal bijkomend gevraagd worden om de RAID-vragenlijst in te vullen in de Sidekick-app. Deze korte vragenlijst peilt naar de lichamelijke en psychische impact van de ziekte aan de hand van slechts zeven vragen. Op basis van een willekeurige, computergestuurde onderverdeling zullen deelnemers deze vragenlijst 1x per week dan wel 1x per maand opgestuurd krijgen. Met behulp van deze gegevens zullen de onderzoekers trachten zich een beter beeld te vormen over hoe de activiteit van de ziekte en de impact hiervan schommelt in de tijd, bijvoorbeeld tussen twee consultaties.

Opmerking: Alle gegevens die u ons als deelnemer bezorgt, zowel via de app als tijdens de consultaties, zullen enkel gebruikt worden voor onderzoeksdoeleinden. Dit is ook opgenomen in het contract dat de onderzoekers aangingen met de fabrikant van de Sidekick-app.

Indien u besluit deel te nemen aan de studie en aan alle voorwaarden voor deelname voldoet, zal u onderstaande testen en onderzoeken doorlopen:

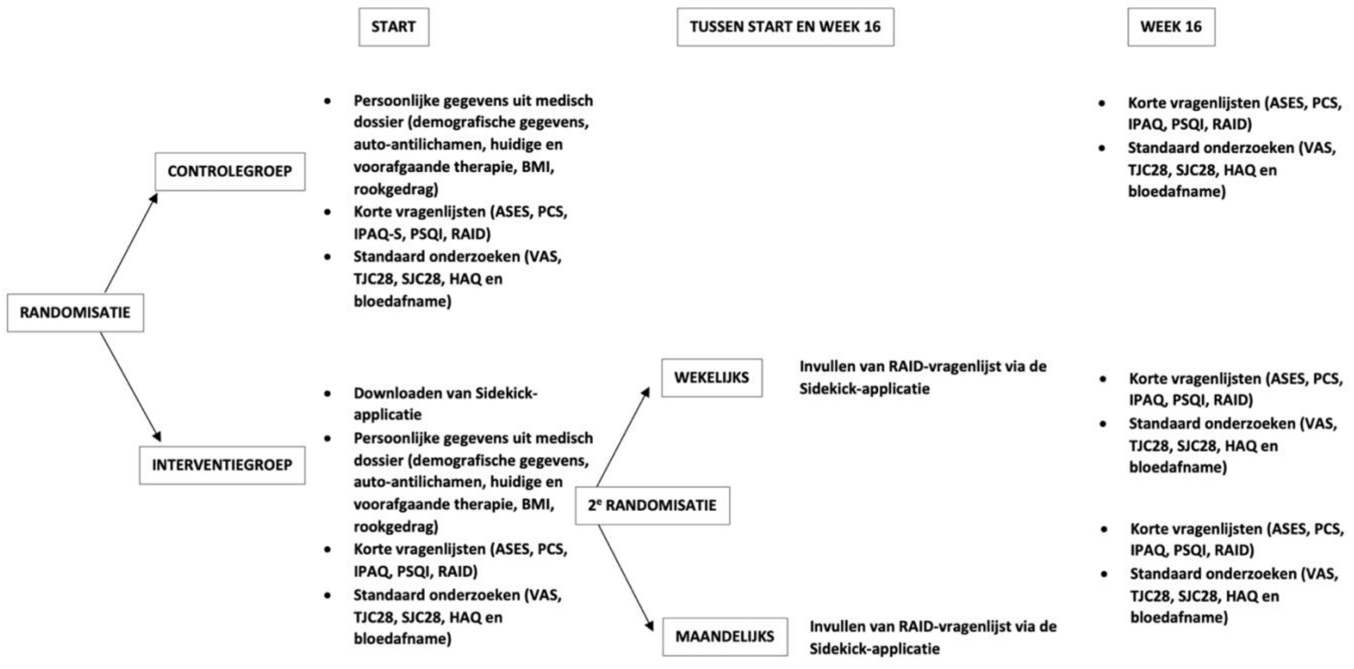

TJC28 = aantal pijnlijke gewrichten, SJC28 = aantal gezwollen gewrichten, VAS = persoonlijke inschatting van de ziekteactiviteit op een schaal van 0-100, HAQ = vragenlijst die peilt naar lichamelijke beperkingen, ASES = vragenlijst die peilt naar zelfredzaamheid, PCS = vragenlijst die peilt naar pijnpercepties, IPAQ = vragenlijst die peilt naar lichaamsbeweging, PSQI = vragenlijst die peilt naar slaapkwaliteit, RAID = vragenlijst die peilt naar impact van de ziekte

Ongemakken

Deelname aan deze klinische studie heeft geen enkele invloed op de gebruikelijke zorg en behandeling die u ontvangt voor uw reumatoïde artritis. Deze studie houdt dan ook geen wijziging van uw medicatie in. Deelname aan de studie biedt naar verwachting dus geen risico op bijkomende fysieke ongemakken of nevenwerkingen.

Privacy en gegevensbescherming

Het onderzoeksteam doet er alles aan om uw persoonsgegevens maximaal te beschermen. Bij deelname aan de studie zal het onderzoeksteam u een uniek gebruikersprofiel toekennen. Dit profiel is verbonden aan een e-mailadres dat specifiek voor de studie aangemaakt werd. Uw eigen e-mailadres en naam blijven dan ook verborgen voor de fabrikant van de app, tenzij u er zelf voor kiest om uw naam of andere persoonsgegevens in het profiel aan te geven. Bij afloop van de studie wordt het gebruikersprofiel hoe dan ook door het onderzoeksteam verwijderd. Op dat moment verdwijnen de gegevens die u als deelnemer invoerde in de app automatisch van de Sidekick-servers, zoals aangegeven in de algemene voorwaarden van de app.

Indien u bijkomende vragen of bezorgdheden heeft over de verwerking van uw persoonsgegevens, kan u steeds contact opnemen met de functionaris voor gegevensbescherming van Sidekick via e-mail naar privacy@sidekickhealth.com.

Voordelen

Indien u besluit om aan deze studie deel te nemen, krijgt u hoe dan ook onbeperkte en volledige toegang tot de inhoud van de mobiele Sidekick-applicatie. Afhankelijk van uw willekeurige groepsindeling krijgt u deze toegang reeds bij de start van de studie (interventiegroep), dan wel pas na afloop (controlegroep). Het gebruik van deze applicatie kan al dan niet gunstig blijken voor het zelfmanagement van uw aandoening, het stimuleren van een gezonde levensstijl of het verminderen van de ziektesymptomen. De informatie die dankzij dit onderzoek verkregen wordt, kan daarnaast bijdragen tot een betere kennis rond het gebruik van mobiele applicaties, ziekte-educatie en motiverend levensstijladvies in de behandeling van reumatoïde artritis bij toekomstige patiënten. Er is evenwel geen garantie dat uw deelname aan deze studie u rechtstreeks voordeel zal opleveren.

Stopzetting van de deelname

Uw deelname is vrijwillig. U hebt het recht om uw deelname aan de studie om eender welke reden en zonder opgave van redenen stop te zetten. Wel kan het voor de arts-onderzoeker en de opdrachtgever nuttig zijn om te weten of u zich terugtrekt omdat de aan de studie verbonden verplichtingen te zwaar zijn.

Ook gebeurt het soms dat de bevoegde nationale of internationale autoriteiten, de ethische comités die aanvankelijk goedkeuring hadden gegeven voor de studie of de opdrachtgever de studie stopzetten omdat bijvoorbeeld uit de verzamelde informatie blijkt dat de interventie niet werkt (de gezondheid van de deelnemers verbetert niet voldoende).

Behandeling na stopzetting van de studie

Indien u aan de volledige studie hebt deelgenomen en dit wenst, kan u de Sidekick-applicatie ook na afloop van de studie gratis en onbeperkt blijven gebruiken. Dit geldt ook wanneer u op willekeurige basis ingedeeld wordt in de controlegroep. Na afloop van de studie kan u uw account bij Sidekick desgewenst ook op elk moment stopzetten, waarna alle persoonlijk identificeerbare gegevens verbonden aan uw account zullen worden verwijderd van de Sidekick-servers.

Biologische stalen die tijdens de studie worden afgenomen

Bij deelname aan deze studie zullen geen bijkomende bloedstalen afgenomen worden ten opzichte van de standaard zorgverlening.

De opdrachtgever van de studie verbindt zich ertoe dat afgenomen stalen uitsluitend gebruikt zullen worden in de context vermeld in de rubriek "Verloop van de klinische studie".

De overschot van de stalen die voor de standaardzorg beschreven in dit document worden afgenomen, zullen niet worden bijgehouden zodra de in dit document beschreven analyses zijn uitgevoerd.

Indien u aan deze studie deelneemt, vragen wij u het volgende:

- Ten volle mee te werken voor een correct verloop van de studie.
- Geen informatie over uw gezondheidstoestand, de applicatie die u gebruikt of de symptomen die u ervaart te verzwijgen.
- De algemene voorwaarden van de applicatie, Sidekick Health, te aanvaarden bij installatie.

U moet eveneens weten dat:

- **De gegevens en vragenlijsten die ingevuld worden in de app niet rechtstreeks worden opgevolgd door het onderzoeksteam tijdens het verloop van de studie. Bij dringende medische problemen vragen we u dan ook zeker de arts te verwittigen.**
- **De gegevens die u invoert in de app niet automatisch opgeslagen worden in uw medisch dossier.**

Contact

Als u bijkomende informatie wenst, maar ook ingeval van problemen of als u zich zorgen maakt, kan u contact opnemen met de arts-onderzoeker (prof. dr. Verschueren, Patrick; patrick.verschueren@uzleuven.be) of een medewerker van zijn/haar studieteam (dr. Doumen, Michaël; michael.doumen@kuleuven.be) via het telefoonnummer (016/342541).

Buiten de consultatie-uren moet u zich aanmelden op de spoedafdeling van uw ziekenhuis en vermelden dat u deelneemt aan een klinische studie. Uw dossier zal nuttige informatie bevatten voor de behandelende arts met betrekking tot de studie.

Als u vragen hebt met betrekking tot uw rechten als deelnemer aan de studie, kan u contact opnemen met de ombudsdienst in uw ziekenhuis op het telefoonnummer: 016/344881. Indien nodig kan de ombudsdienst u in contact brengen met het Ethisch Comité.

Bij vragen of bezorgdheden rond privacy of GDPR-aangelegenheden kan u contact opnemen met de data protection officer (DPA) van het Universitair Ziekenhuis Leuven via dpo@uzleuven.be, en met de functionaris voor gegevensbescherming van Sidekick via e-mail naar privacy@sidekickhealth.com.

Titel van de studie: De invloed van ziekte-educatie en doelgericht levensstijladvies, aangeboden via een mobiele app, op zelfredzaamheid en een gezonde levensstijl bij patiënten met reumatoïde artritis.

II Geïnformeerde toestemming

Deelnemer

Ik verklaar dat ik geïnformeerd ben over de aard, het doel, de duur, de eventuele voordelen en risico's van de studie en dat ik weet wat van mij wordt verwacht. Ik heb kennis genomen van het informatiedocument en de bijlagen ervan.

Ik heb voldoende tijd gehad om na te denken en desgewenst met een door mij gekozen persoon, zoals mijn huisarts of een familielid, te praten.

Ik heb alle vragen kunnen stellen die bij me opkwamen en ik heb een duidelijk antwoord gekregen op mijn vragen.

Ik begrijp dat mijn deelname aan deze studie vrijwillig is en dat ik vrij ben mijn deelname aan deze studie stop te zetten zonder dat dit mijn relatie schaadt met het therapeutisch team dat instaat voor mijn gezondheid.

Ik begrijp dat er tijdens mijn deelname aan deze studie gegevens over mij zullen worden verzameld en dat de arts-onderzoeker en de opdrachtgever de vertrouwelijkheid van deze gegevens verzekeren overeenkomstig de Belgische wetgeving ter zake. Ik begrijp dat het uitvoeren van deze studie door UZ Leuven het algemeen belang dient en de verwerking van mijn persoonsgegevens noodzakelijk is voor het uitvoeren van deze studie.

Mijn huisarts en andere specialisten die betrokken zijn bij mijn behandeling zullen op de hoogte worden gesteld van mijn deelname aan deze klinische studie.

Ik heb een exemplaar ontvangen van de informatie aan de deelnemer en de geïnformeerde toestemming.

Naam, voornaam, datum en handtekening van de deelnemer

Arts-onderzoeker

Ik ondergetekende ……………………………………………………………..…, arts-onderzoeker / bevoegde onderzoeksmederwerker, verklaar de benodigde informatie inzake deze studie mondeling te hebben verstrekt evenals een exemplaar van het informatiedocument aan de deelnemer te hebben verstrekt.

Ik bevestig dat geen enkele druk op de deelnemer is uitgeoefend om hem/haar te doen toestemmen tot deelname aan de studie en ik ben bereid om op alle eventuele bijkomende vragen te antwoorden.

Ik bevestig dat ik werk in overeenstemming met de ethische beginselen zoals vermeld in de laatste versie van de "Verklaring van Helsinki", de "Goede klinische praktijk" en de Belgische wet van 7 mei 2004 inzake experimenten op de menselijke persoon.

Naam, Voornaam, Datum en handtekening Naam, Voornaam, Datum en handtekening
van de vertegenwoordiger van de arts-onderzoeker
van de arts-onderzoeker

Titel van de studie: De invloed van ziekte-educatie en doelgericht levensstijladvies, aangeboden via een mobiele app, op zelfredzaamheid en een gezonde levensstijl bij patiënten met reumatoïde artritis.

III Aanvullende informatie

1 : Risico's in verband met de klinische onderzoeksprocedures

Bij deelname aan deze studie zullen geen bijkomende bloedstalen afgenomen worden ten opzichte van de standaard zorgverlening. Wanneer een bloedafname nodig is in kader van routine zorg, kan deze (in zeldzame gevallen) pijn, bloedingen, bloeduitstortingen of een lokale infectie op de plek van bloedafname veroorzaken. Ook kunnen sommige deelnemers zich duizelig voelen of flauwvallen tijdens de afname. Het personeel dat de bloedafname uitvoert, zal alles in het werk stellen om deze ongemakken te beperken.

2 : Aanvullende informatie over de bescherming en de rechten van deelnemers aan een klinische studie

Ethische comités

Deze studie werd geëvalueerd door een onafhankelijk ethisch comité [Ethisch comité onderzoek UZ/KU Leuven] dat een gunstig advies heeft uitgebracht [*na raadpleging van de Ethische Comités van elk centrum waarin deze studie zal worden uitgevoerd*]. De ethische comités hebben als taak de personen die aan klinische studies deelnemen te beschermen. Ze controleren of uw rechten als patiënt en als deelnemer aan een studie gerespecteerd worden, of - uitgaande van de huidige kennis - de balans tussen risico's en voordelen gunstig is voor de deelnemers, of de studie wetenschappelijk relevant en ethisch verantwoord is.

Hierover brengen de ethische comités een advies uit in overeenstemming met de Belgische wet van 7 mei 2004.

U dient het positief advies van de Ethische Comités in geen geval te beschouwen als een aansporing om deel te nemen aan deze studie.

Vrijwillige deelname

Aarzel niet om alle vragen te stellen die bij u opkomen voordat u tekent. Neem de tijd om er over te praten met een vertrouwenspersoon indien u dat wenst.

U heeft het recht om niet deel te nemen aan deze studie of met deze studie te stoppen, zonder dat u hiervoor een reden hoeft te geven, zelfs al hebt u eerder toegestemd om aan deze studie deel te nemen. Uw beslissing zal in geen geval uw relatie met de arts-onderzoeker beïnvloeden, noch de kwaliteit van uw verdere verzorging.

Als u aanvaardt om aan deze studie deel te nemen, ondertekent u het toestemmingsformulier. De arts-onderzoeker zal dit formulier ook ondertekenen en zal zo bevestigen dat hij u de noodzakelijke informatie over deze studie heeft gegeven. U zal het voor u bestemde exemplaar ontvangen.

Voor uw veiligheid is het wel aanbevolen om de arts-onderzoeker op de hoogte te stellen indien u besluit uw deelname aan de studie stop te zetten.

Kosten in verband met uw deelname

Indien u besluit om aan deze studie deel te nemen, brengt dit geen bijkomende kosten met zich mee voor u of voor uw verzekeringsmaatschappij. De kosten verbonden aan

de studievisites worden terugbetaald via het gebruikelijke terugbetalingssysteem van RIZIV/INAMI. Alleen kosten in verband met gebruikelijke medische prestaties in uw klinische situatie, kunnen u aangerekend worden.

De kosten van de onderzochte behandeling (in dit geval de volledige versie van de Sidekick-applicatie) zijn volledig ten laste van de opdrachtgever.

Vertrouwelijkheidsgarantie

Uw deelname aan de studie betekent dat de arts-onderzoeker gegevens over u verzamelt en dat de opdrachtgever van de studie die gebruikt voor onderzoek en in het kader van wetenschappelijke en medische publicaties. De verwerking van uw persoonsgegevens is noodzakelijk om de wetenschappelijke onderzoeksdoeleinden zoals hierin te kunnen realiseren. Het uitvoeren van academische onderzoek behoort tot wettelijke opdrachten van UZ Leuven als opdrachtgever. Als universitair ziekenhuis verbonden aan de KU Leuven dient UZ Leuven immers wetenschap en onderwijs in het algemeen belang te ondersteunen. UZ Leuven verduidelijkt u graag dat de noodzakelijkheid van de verwerking voor het uitvoeren van wetenschappelijk onderzoek en dit als taak van algemeen belang, de wettelijke toelatingsgrond vormt op basis waarvan UZ Leuven in het kader van dit onderzoek uw gegevens verwerkt. Daarnaast is UZ Leuven onderhevig aan specifieke wettelijke verplichtingen die de verwerking van uw gegevens mogelijks noodzakelijk maken in het kader van veiligheidsrapportering (zoals bijvoorbeeld het melden van bijwerkingen aan toezichthoudende overheidsinstanties).

U hebt het recht om aan de arts-onderzoeker te vragen welke gegevens hij/zij over u heeft verzameld en waarvoor ze gebruikt worden in het kader van de studie. Deze gegevens hebben betrekking op uw huidige klinische situatie maar ook op uw medische voorgeschiedenis en op de resultaten van onderzoeken die werden uitgevoerd voor de behandeling van uw gezondheid volgens de geldende zorgstandaard. U hebt het recht om deze gegevens in te kijken en om verbeteringen te laten aanbrengen indien ze foutief zouden zijn[2].

De arts-onderzoeker is verplicht om deze verzamelde gegevens vertrouwelijk te behandelen.

Dit betekent dat hij/zij zich ertoe verbindt om uw naam nooit bekend te maken bv in het kader van een publicatie of een conferentie en dat hij/zij uw gegevens zal coderen (uw identiteit zal worden vervangen door een identificatiecode in de studie) voordat hij/zij ze doorgeeft aan de beheerder van de databank (UZ Leuven Dienst Reumatologie, Datamanager: Johan Joly).

De arts-onderzoeker en zijn team zullen gedurende de volledige klinische studie de enige personen zijn die een verband kunnen leggen tussen de overgedragen gegevens en uw medisch dossier[3].

De overgedragen persoonlijke gegevens omvatten geen combinatie van elementen waarmee het mogelijk is u te identificeren[4].

[2] Deze rechten zijn bepaald door de wet van 8 december 1992 tot bescherming van de persoonlijke levenssfeer ten opzichte van de verwerking van persoonsgegevens en door de wet van 22 augustus 2002 betreffende de rechten van de patiënt.

[3] Voor klinische studies verplicht de wet om het verband met uw dossier gedurende 20 jaar te behouden. In geval van een studiegeneesmiddel voor een innoverende therapie waarbij gebruik wordt gemaakt van menselijk lichaamsmateriaal, bedraagt deze periode minimaal 30 jaar en maximaal 50 jaar in overeenstemming met de Belgische wet van 19 december 2008 inzake het gebruik van menselijk lichaamsmateriaal en de geldende Koninklijke Besluiten.

[4] De gegevensbank met onderzoeksresultaten bevat dus geen verband met elementen zoals uw initialen, uw geslacht en uw volledige geboortedatum (dd/mm/jjjj).

De door de opdrachtgever aangestelde beheerder van de onderzoeksgegevens kan u niet identificeren op basis van de overgedragen gegevens. Deze persoon is verantwoordelijk voor het verzamelen van de gegevens die door alle artsen-onderzoekers die deelnemen aan de studie zijn verzameld en voor de verwerking en de bescherming van die gegevens in overeenstemming met de Belgische wet betreffende de bescherming van de persoonlijke levenssfeer.

Om de kwaliteit van de studie te controleren, kan uw medisch dossier worden ingekeken door personen die gebonden zijn aan het beroepsgeheim zoals vertegenwoordigers van de ethische comités, van de opdrachtgever van de studie of een extern auditbureau. Dit kan enkel gebeuren onder strikte voorwaarden, onder de verantwoordelijkheid van de arts-onderzoeker en onder zijn/haar toezicht (of van één van zijn/haar onderzoeksmedewerkers).

De (gecodeerde) onderzoeksgegevens kunnen doorgegeven worden aan Belgische of andere regelgevende instanties, aan de betrokken ethische comités, aan andere artsen en/of instellingen die samenwerken met de opdrachtgever.

Ze kunnen ook doorgegeven worden aan andere sites van de opdrachtgever in België en in andere landen waar de normen inzake de bescherming van persoonsgegevens verschillend of minder strikt kunnen zijn[5]. Dit gebeurt dan steeds in gecodeerde vorm zoals hierboven uitgelegd.

Uw toestemming om aan deze studie deel te nemen betekent dus ook dat uw gecodeerde medische gegevens gebruikt worden voor doeleinden die in dit informatieformulier beschreven staan en dat ze overgedragen worden aan bovenvermelde personen en/of instellingen.

De opdrachtgever zal de verzamelde gegevens gebruiken in het kader van de studie waaraan u deelneemt.

Indien u uw toestemming tot deelname aan de studie intrekt, zullen de gecodeerde gegevens die al verzameld waren vóór uw terugtrekking, bewaard worden. Hierdoor wordt de geldigheid van de studie gegarandeerd. Er zal geen enkel nieuw gegeven aan de opdrachtgever worden doorgegeven.

Wat gebeurt er met uw staal/stalen tijdens en na afloop van de studie?

Er worden geen extra stalen afgenomen voor de studie, de afgenomen stalen maken deel uit van de routinezorg.

Verzekering

Elke deelname aan een studie houdt een risico in, hoe klein ook. De opdrachtgever is - ook indien er geen sprake is van fout - aansprakelijk voor de schade die de deelnemer of in geval van overlijden zijn/haar rechthebbenden, oplopen en die rechtstreeks of onrechtstreeks verband houdt met diens deelname aan de studie. U moet hiervoor dus geen fout aantonen. De opdrachtgever heeft voor deze aansprakelijkheid een verzekering afgesloten[6].

We verzoeken u daarom om elk nieuw gezondheidsprobleem aan de arts-onderzoeker te melden. Hij/Zij kan u aanvullende informatie verstrekken over mogelijke behandelingen. En bij dringende medische problemen vragen we u dan ook zeker de arts te verwittigen. Indien de arts-onderzoeker van mening is dat er een verband met de studie mogelijk is (er is geen verband met de studie bij schade ten gevolge van het

---

[5] De opdrachtgever verbindt er zich toe om de voorwaarden in de Europese Richtlijnen en de Belgische Wetgeving betreffende de bescherming van de persoonlijke levenssfeer te eerbiedigen.

[6] In overeenstemming met artikel 29 van de Belgische Wet inzake experimenten op de menselijke persoon (7 mei 2004)

natuurlijke verloop van uw ziekte of ten gevolge van gekende bijwerkingen van uw standaardbehandeling), zal hij/zij de opdrachtgever van de studie op de hoogte stellen die de aangifteprocedure bij de verzekering zal starten. Deze zal, indien zij het nodig acht, een expert aanstellen om een oordeel uit te spreken over het verband tussen uw nieuwe gezondheidsklachten en de studie.

In het geval van onenigheid met de arts-onderzoeker of met de door de verzekeringsmaatschappij aangestelde expert, en steeds wanneer u dit nodig acht, kunnen u of in geval van overlijden uw rechthebbenden de verzekeraar rechtstreeks in België dagvaarden (Amlin Insurance SE, 299.053.700, contactgegevens makelaar: Vanbreda Risk & Benefits NV, Plantin en Moretuslei 297, 2140 Antwerpen).
